# Detecting Starch-Head and Mildewed Fruit in Dried Hami Jujubes Using Visible/Near-Infrared Spectroscopy Combined with MRSA-SVM and Oversampling

**DOI:** 10.3390/foods11162431

**Published:** 2022-08-12

**Authors:** Yujie Li, Benxue Ma, Yating Hu, Guowei Yu, Yuanjia Zhang

**Affiliations:** 1 College of Mechanical and Electrical Engineering, Shihezi University, Shihezi 832003, China; 2 Key Laboratory of Northwest Agricultural Equipment, Ministry of Agriculture and Rural Affairs, Shihezi 832003, China

**Keywords:** dried Hami jujube, visible/near-infrared spectroscopy, defective fruit detection, reptile search algorithm, oversampling technique, non-destructive detection

## Abstract

Dried Hami jujube has great commercial and nutritional value. Starch-head and mildewed fruit are defective jujubes that pose a threat to consumer health. A novel method for detecting starch-head and mildewed fruit in dried Hami jujubes with visible/near-infrared spectroscopy was proposed. For this, the diffuse reflectance spectra in the range of 400–1100 nm of dried Hami jujubes were obtained. Borderline synthetic minority oversampling technology (BL-SMOTE) was applied to solve the problem of imbalanced sample distribution, and its effectiveness was demonstrated compared to other methods. Then, the feature variables selected by competitive adaptive reweighted sampling (CARS) were used as the input to establish the support vector machine (SVM) classification model. The parameters of SVM were optimized by the modified reptile search algorithm (MRSA). In MRSA, Tent chaotic mapping and the Gaussian random walk strategy were used to improve the optimization ability of the original reptile search algorithm (RSA). The final results showed that the MRSA-SVM method combined with BL-SMOTE had the best classification performance, and the detection accuracy reached 97.22%. In addition, the recall, precision, F_1_ and kappa coefficient outperform other models. Furthermore, this study provided a valuable reference for the detection of defective fruit in other fruits.

## 1. Introduction

Dried Hami jujube is deeply loved by consumers and is a best-selling product in domestic and foreign markets [1]. During the processing and storage process of dried jujubes, starch-head and mildewed fruit are prone to be affected by the level of drying technology and the storage environment [2]. If the defective fruit is not removed in time, the grade of Hami jujube will decline. Mildewed jujube contains mycotoxins, which can cause food poisoning and threaten the health of consumers. Due to the influence of moisture, the starch-head jujube will further develop into mildewed jujube during the storage process, leaving food safety hazards. The rapid and non-destructive detection of starch-head and mildewed fruit in dried Hami jujubes is of great significance to ensure food safety and safeguard the interests of consumers.

Manual visual inspection and machine vision methods are commonly used in the defective fruit detection of dried jujubes [3,4]. Manual visual inspection is highly subjective and inefficient. Although machine vision can detect defective fruit, it can only identify fruit with obvious changes in surface morphology and color. In addition, image information can only reflect physical properties of dried jujubes, but it cannot provide information on the chemical properties. In practice, both physical and chemical properties are important indicators for the quality detection of dried red dates, which can characterize their defect information. Visible/near-infrared (Vis-NIR) spectroscopy is a fast, non-destructive and high-sensitivity detection method [5], which can simultaneously obtain the physical and chemical information of the measured object, and it has been applied in the field of quality detection of dried jujubes [6,7]. Some studies applied spectroscopy to detect defective fruit in peanuts [8], chestnuts [9], almond kernels [10] and macadamia nuts [11]. There are few studies on the non-destructive detection of mildewed fruit in jujubes by NIR spectroscopy [12,13], and there is no report on the detection of starch-head fruit.. Hence, one of the goals of this study is to explore the potential of Vis-NIR spectroscopy to identify starch-head and mildewed fruit.

Machine learning (ML) has penetrated into the field of spectral analysis. Support vector machines (SVM), partial least squares (PLS), k-nearest neighbors (KNN), artificial neural networks (ANN), etc. have shown good potential in quality evaluation [14]. Nevertheless, data imbalance can trigger inaccurate analysis in ML classification. The sample size of the minority category is less than that of the majority category, which leads the model to favor the latter and ignore the former in pursuit of global accuracy [15]. Oversampling methods are often applied to solve data imbalance problems, such as synthetic minority over-sampling technology (SMOTE) and its improved algorithms. Amirruddin et al. [16] integrated SMOTE with logistic model tree adaptive boosting for assessing nutrient and chlorophyll sufficiency levels of oil palm and demonstrated that SMOTE can provide classification accuracy. Begum et al. [17] used the oversampling algorithm to process imbalance data in coal samples and demonstrated its effectiveness. Similarly, the oversampling algorithms were used for the detection of moldy apple core [18]. Still, few studies have focused on the effects of different oversampling strategies on the classification performance of the model. Thus, various oversampling methods were introduced and compared to detect starch-head and mildewed fruit in dried Hami jujubes. In addition, the most suitable oversampling method was determined.

The parameters of ML models are mostly set using empirical settings or grid searches (GS), which have the shortcomings of being highly subjective and consuming computational resources. Meta-heuristic algorithms are one of the most popular optimization approaches that perform global and local searches for a specific problem by simulating relevant behaviors in biology, physics, and other fields to achieve optimization. The use of meta-heuristic algorithms for the parameter optimization of ML models is an idea of powerful combination that can fully integrate the advantages of both and expand the application scope of the models. More and more meta-heuristic algorithms have been developed in the field of spectral analysis, such as genetic algorithm (GA) [7], particle swarm optimization algorithm (PSO) [19], gray wolf optimizer algorithm (GWO) [20], marine predators algorithm (MPA) [21] and so on. Reptile search algorithm (RSA) is a novel meta-heuristic algorithm inspired by the group hunting patterns of crocodile populations [22]. RSA has obvious advantages such as strong robustness, fast convergence speed and good optimization effect. However, as with other meta-heuristic algorithms, the population diversity of RSA decreases when the search approaches the global optimum, and it is easy to fall into a local optimum solution. So, this paper attempts to improve the RSA by introducing chaotic initialization and Gaussian random walking strategy. Then, the modified RSA was applied in the parameter optimization of the ML model in order to improve the detection accuracy of starch-head and mildewed fruit in dried Hami jujubes.

The specific objectives of this study were the following: (1) to obtain Vis-NIR spectra of dried Hami jujube and determine chemometrics for defective jujube detection; (2) to evaluate the impact of different oversampling methods on classification models and determine the most suitable strategy; (3) to propose a modified reptile search algorithm (MRSA) and use it for parameter optimization of machine learning models; and (4) to establish classification models based on different meta-heuristic algorithms and compare their performance.

## 2. Materials and Methods

### 2.1. Sample Preparation

**Figure 1:**
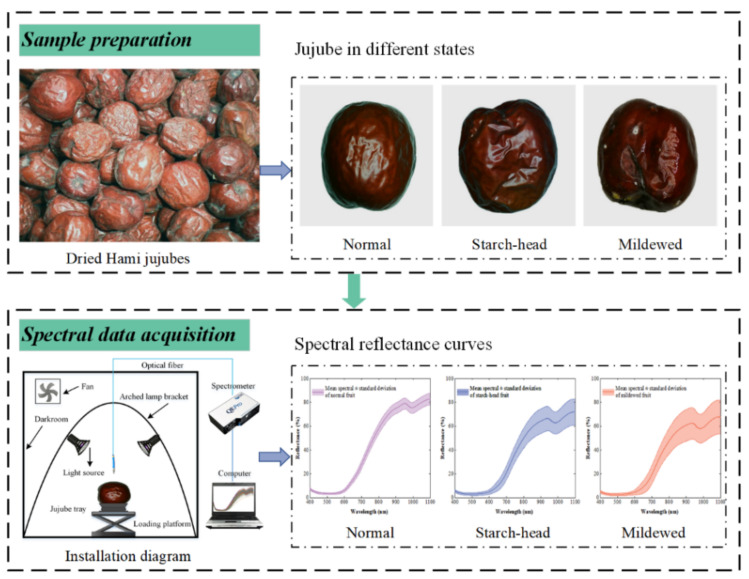
Dried Hami jujube samples and spectral data acquisition.

The same batch of naturally dried Hami jujubes with moisture content of about 28% was purchased from an agricultural market in Hami, China and transported to Shihezi University for sample preparation. A total of 600 dried Hami jujubes with similar color, shape, and weight, without apparent defects, physical injuries or disease infection were selected. Each sample surface was wiped with 75% ethanol aqueous solution, distilled water for 10 s, and then air-dried. According to the reasons for the formation of starch-head jujube and mildewed jujube during the drying and storing process [2], the corresponding environment was simulated to prepare samples in different states. Four hundred samples were randomly selected, 2 mL of distilled water was injected by a micro syringe into the subcutaneous 5 mm of each sample through the calyx shoulder region and then stored in a thermostatic container kept at 30 °C and 85% relative humidity (RH) for 7 days. The remaining 200 dried Hami jujube samples were directly placed in another thermostatic container kept at 25 °C and 40% RH, which were regarded as normal jujubes. The state of the dried Hami jujube was determined according to the comprehensive conditions of the appearance, fruit shape, texture and smell. Jujube samples in different states are shown in Figure 1. Normal jujube: the peel is purple-red and shiny, the fruit shape is plump, the color of pulp is light yellow and uniform, the pulp is firm, and it has a strong fruit aroma. Starch-head fruit: the peel is slightly discolored, the local moisture content is high, the pulp is rotten, inelastic or even pus-like, and it will further develop into mildewed fruit. The starch-head fruit was hard to identify by the naked eye directly. Mildewed fruit: the peel color appears dull, the pulp is deteriorated and emits wine or a musty odor, and mycelium appear on its surface. It should be noted that in this study, mildew degree was slight, that is, only a small amount of mycelium appeared in the mildewed jujube. In the end, 200 normal jujubes, 302 starch-head fruit, and 98 mildewed fruit were obtained, respectively. Samples were then separated into the training set and test set based on a 7:3 ratio. Among them, 420 dried Hami jujubes were used as the training set (including 140 normal jujubes, 211 starch-head fruit and 69 mildewed fruit) to establish the classification model, and the remaining 180 samples constituted the test set (including 60 normal jujubes, 91 starch-head fruit and 29 mildewed fruit) to evaluate the performance of the model. The training set and the test set were completely independent to better evaluate the robustness of the model.

### 2.2. Spectral Data Acquisition

A dried Hami jujube diffuse reflection spectrum acquisition system based high performance-spectrometer was developed independently, as shown in Figure 1; it comprised the following: a high performance-spectrometer (QE Pro-FL, Ocean Insight, USA), 600 μm diameter optical fiber (QP600-2-VIS-NIR, Ocean Insight, USA), halogen light source (rated voltage of 12 V, power of 20 W), arched lamp bracket, a jujube tray, loading platform, fan, darkroom and computer. The halogen light sources were fixed on arched lamp bracket, and the lighting angle was about 60°. We kept the optical fiber probe perpendicular to the sample and about 40 mm from the surface of the dried Hami jujube. The acquisition range of the high-performance-spectrometer was 348–1141 nm, with a total of 1044 bands. We turned on the spectrometer and light source to warm up for 30 min before operation. We also set appropriate parameters of the spectrometer and used white and dark references for correction. In particular, the integration time was set to 121 ms, the moving smoothing width was set to 4, and the average number of scans was set to 8. At the equatorial plane of each sample, four positions were uniformly selected (90° apart) to collect diffuse reflection spectral information, and the average value of four data points was taken as the original spectral of the jujube.

### 2.3. Spectral Data Processing

#### 2.3.1. Spectral Pre-Processing

The raw spectral data usually carry some information and noise irrelevant to sample composition, which will affect the correspondence between spectral information and classification labels, and then affect the stability of the classification model. It is necessary to pre-process raw spectra before modeling. All raw spectral data were pre-processed by multiple scatter correction [23].

#### 2.3.2. Handling of Class Imbalance

The classification accuracy depends on the amount and quality of the modeled dataset in machine learning models. In this study, a total spectral data of 600 dried Hami jujubes were obtained, and the number of different states was 200 for normal jujube (33.3%), 302 for starch-head fruit (50.4%), and 98 for mildewed fruit (16.3%), respectively. Obviously, the data distribution was imbalanced. This imbalance persisted after randomly splitting the spectral data into training and test sets. Hence, an oversampling technique was applied to handle the imbalance in these data during training. We analyzed four frequently used approaches for data augmentation, namely random oversampling (ROS), synthetic minority oversampling technique (SMOTE) [24], borderline synthetic minority oversampling technique (BL-SMOTE) [25] and adaptive synthetic sampling (ADASYN) [26], and we compared them to the model without any data augmentation.

The ROS method randomly replicates samples from the minority class and increases them to the training data to alleviate the imbalance between samples. The essential idea of SMOTE is to synthesize new samples by linear interpolation based on the KNN. The BL-SMOTE is another oversampling method improved by SMOTE. We calculated the *k*-nearest neighbor of the minority class samples and divided them into noise, safe and danger categories according to the distribution densities. Finally, we used random linear interpolation to generate new samples only for danger samples. The ADASYN works similarly to SMOTE, but it focuses more on sample synthesis at the boundaries of the minority and majority classes. This method uses weighted distribution to adaptively synthesize minority class samples according to the learning difficulty level, where more synthetic data are generated for difficult-to-learn samples. The number of *k*-nearest neighbors for all approaches was set to 5.

#### 2.3.3. Variable Selection Strategy

Redundant variables in the raw spectral data complicate the classification model and affect the accuracy and stability of the model. Thus, appropriate variable selection strategies are necessary. In this study, competitive adaptive reweighted sampling (CARS), successive projections algorithm (SPA) and iteratively retains informative variables (IRIV) were used to identify the effective variables from full spectra. For CARS, the number of Monte Carlo sampling runs was set to 50, and five-fold cross-validation was applied to determine the optimal spectra set. For SPA, we set the variable selection range to 5–20 and the selection step to 1. As for IRIV, the maximum principal component and the number of cross-validation was 15 and 5, respectively. The specific steps of algorithms can refer to previous studies [27,28,29] and will not be described in detail here.

### 2.4. Support Vector Machine Based on Modified Reptile Search Algorithm

#### 2.4.1. Support Vector Machine (SVM)

SVM is based on VC dimension theory and the structural risk minimization principle, which can solve the problem of constructing high-dimensional models with a limited number of samples [30]. A radial basis function (RBF) with good stability was introduced as the kernel function for operation. In the SVM model, the penalty parameter *c* and the kernel parameter *g* are extremely important and were determined by grid search. The main function of penalty parameter *c* is to control the tolerance limit of credibility, in other words, to determine the model’s tolerance for wrong samples. The kernel parameter *g* determines the form of the classification hyperplane. Determining the optimal combination of *c* and *g* within a certain range can improve the classification accuracy of SVM.

#### 2.4.2. Reptile Search Algorithm (RSA)

The RSA is a novel meta-heuristic algorithm to solve complex optimization and engineering problems. The main idea of RSA comes from the behavior of crocodiles in nature to hunt prey. Crocodiles continue to approach the prey through the encircling phase and the hunting phase until the prey is captured. Key processes of the RSA can be summarized as follows:

(1) Parameter initialization.

Several parameters are set: the number of candidate solutions (*N*); the dimension size of the search space (*D*); the upper bound (*ub*) and lower bound (*lb*) of the solution; and the maximum number of iterations (*T*). So, the initial candidate solutions can be described as shown in Equation (1).

(1) $$X=\left[\begin{array}{ccccc} x_{\left(1,1\right)} & \cdots & x_{\left(1,j\right)} & x_{\left(1,D-1\right)} & x_{\left(1,D\right)}\\ x_{\left(2,1\right)} & \cdots & x_{\left(2,j\right)} & \cdots & x_{\left(2,D\right)}\\ \cdots & \cdots & x_{\left(i,j\right)} & \cdots & \cdots \\ \vdots & \vdots & \vdots & \vdots & \vdots \\ x_{\left(N-1,1\right)} & \cdots & x_{\left(N-1,j\right)} & \cdots & x_{\left(N-1,D\right)}\\ x_{\left(N,1\right)} & \cdots & x_{\left(N,j\right)} & x_{\left(N,D-1\right)} & x_{\left(N,D\right)} \end{array}\right]$$

where *X* is a set of the candidate solutions that are randomly generated using Equation (2), and $x_{\left(i,j\right)}$ denotes the *j_th_* position of the *i_th_* solution.

(2) $$x_{\left(i,j\right)}=rand_{\in \left[0,1\right]}\times \left(ub-lb\right)+lb\;\;\;i\in \left\{1,\ldots ,N\right\}\;and\;j\in \left\{1,\ldots ,D\right\}$$

(2) Encircling phase (exploration).

The purpose of the encircling phase is to perform global search, which includes two actions strategy: high walking and belly walking. The position update in this phase can be calculated by

(3) $$x_{\left(i,j\right)}\left(t+1\right)=\left\{\begin{array}{l} Best_{j}\left(t\right)\times -\eta_{\left(i,j\right)}\left(t\right)\times \beta -R_{\left(i,j\right)}\left(t\right)\times rand_{\in \left[0,1\right]}\;\;t\leq \frac{T}{4}\\ Best_{j}\left(t\right)\times x_{\left(r_{1},j\right)}\times ES\left(t\right)\times rand_{\in \left[0,1\right]}\;\;\;t\leq 2\frac{T}{4}\;and\;t>\frac{T}{4} \end{array}\right.$$

where $Best_{j}\left(t\right)$ is the *j_th_* position in the best-obtained solution so far; *t* is the number of the current iteration; $\eta_{\left(i,j\right)}$ denotes the hunting operator for the *j_th_* position in the *i_th_* solution; *β* is a sensitive parameter to control the exploration accuracy for the encircling phase, which is fixed equal to 0.1; reduce function $R_{\left(i,j\right)}$ is a value used to reduce the search area; *r_1_* is a random number between $\left[1,N\right]$ and $x_{\left(r_{1},j\right)}$ denotes a random position of the *i_th_* solution; Evolutionary Sense ($ES\left(t\right)$) is a probability ratio that takes randomly decreasing values between $\left[1,-2\right]$ throughout the number of iterations. Equations (4)–(6) show the calculation methods of $R_{\left(i,j\right)}$, $ES\left(t\right)$ and $\eta_{\left(i,j\right)}$.

(4) $$R_{\left(i,j\right)}=\frac{Best_{j}\left(t\right)-x_{\left(r_{2},j\right)}}{Best_{j}\left(t\right)+\epsilon }$$

(5) $$ES\left(t\right)=2\times r_{3}\times \left(1-\frac{1}{T}\right)$$

(6) $$\eta_{\left(i,j\right)}=Best_{j}\left(t\right)\times P_{\left(i,j\right)}$$

where *r*_2_ is a random number between $\left[1,N\right]$ while *r*_3_ is a random integer number between $\left[-1,1\right]$. In addition, ϵ denotes a small value to ensure that the denominator is not zero. In Equation (6), $P_{\left(i,j\right)}$ is the percentage difference between the *j_th_* value of the best solution and its corresponding position in the current solution. It is defined as:

(7) $$P_{\left(i,j\right)}=\alpha +\frac{x_{\left(i,j\right)}-M\left(x_{i}\right)}{Best_{j}\left(t\right)\times \left(ub_{\left(j\right)}-lb_{\left(j\right)}\right)+\epsilon }$$

where $ub_{\left(j\right)}$ and $lb_{\left(j\right)}$ are the upper and lower boundaries of the *j_th_* position. α is an another sensitive parameter, which is fixed equal to 0.1 in this paper. $M\left(x_{i}\right)$ stands for the average solutions.

(3) Hunting phase (exploitation).

The purpose of the hunting phase is to perform local search, which includes two actions strategy: coordination and cooperation. The position update in this phase can be calculated by

(8) $$x_{\left(i,j\right)}\left(t+1\right)=\left\{\begin{array}{ll} Best_{j}\left(t\right)\times P_{\left(i,j\right)}\left(t\right)\times rand_{\in \left[0,1\right]} & t\leq 3\frac{T}{4}\;and\;t>2\frac{T}{4}\\ Best_{j}\left(t\right)-\eta_{\left(i,j\right)}\left(t\right)\times \epsilon -R_{\left(i,j\right)}\left(t\right)\times rand_{\in \left[0,1\right]} & t\leq T\;and\;t>3\frac{T}{4} \end{array}\right.$$

In this equation, variables are defined and calculated in the same way as in Equation (3).

The standard RSA’ pseudo-code is provided in [22].

#### 2.4.3. Modified Reptile Search Algorithm (MRSA)

This part presents the details of the MRSA and how it enhances the population diversity and solution accuracy. In addition, this section explains how to prevent being trapped at local optimal solutions. In MRSA, chaotic mapping and Gaussian random walk strategies are used, which can guarantee to alleviate the issues of declining population diversity, falling into a local optimal solution and slow convergence more effectively.

(1) Tent chaotic mapping.

The location of the population initialization can directly affect the convergence speed and solution quality of the algorithm. The RSA algorithm usually uses randomly generated data as the initial population information, which will make it difficult to preserve the diversity of the population and result in poor optimization results of the algorithm. Chaotic sequences have the characteristics of randomness, regularity and ergodicity [31], which can increase the diversity of the initial population and improve the global search ability. In this study, Tent mapping is adopted to initialize the population due to its traversal uniformity and fast iteration speed. Equations (9) and (10) show the calculation method of the Tent chaotic sequence and initial location of the population.

(9) $$z_{i+1}=\left\{\begin{array}{ll} 2\times z_{i} & 0\leq z_{i}\leq 1/2\\ 2\times \left(1-z_{i}\right) & 1/2<z_{i}\leq 1 \end{array}\right.$$

(10) $$x_{\left(i,j\right)}=z_{i,j}\times \left(ub-lb\right)+lb\;\;i\in \left\{1,\ldots ,N\right\}\;and\;j\in \left\{1,\ldots ,D\right\}$$

where $z_{i}$ is the *i_th_* chaotic sequence and $z_{i,j}$ denotes the *j_th_* chaotic value in the $z_{i}$ sequence. In addition, $z_{1}$ is the 1×D dimensional vectors generated by uniform random numbers in $\left[0,1\right)$.

(2) Gaussian random walk strategy.

In the later stage of the iterative process, the swarm diversity of RSA is reduced, and it is easy to converge prematurely. In order to reduce the probability of prematurity and local optimal probability, the Gaussian random walk strategy (GRW) was introduced in this paper, which was applied after the search process. The GRW uses the current optimal individual to adjust the positions of all individuals, thereby increasing the disturbance to the whole population and enhancing the ability of the algorithm to jump out of the local optimum [32]. The GRW based on the current optimal individual is shown in Equations (11) and (12).

(11) $$GX_{i}^{G}=Guassian\left(X_{best}^{G},\tau \right)+\left(g_{1}\cdot X_{best}^{G}-g_{2}\cdot X_{i}^{G}\right)$$

(12) $$\tau =\frac{log\left(G\right)}{G}\left(X_{i}^{G}-X_{best}^{G}\right)$$

where $X_{i}^{G}$ is the *i_th_* individual in iteration *G*, $X_{best}^{G}$ is the best individual in iteration *G*, and $GX_{i}^{G}$ represents new individuals generated after GRW. $g_{1}$ and $g_{2}$ are uniformly distributed random numbers in $\left[0,1\right]$. τ is the step length of random walk, which negatively correlated with the number of iterations.

Through the guidance of the current optimal individual, a new population is generated near the optimal individual, which is conducive to the rapid convergence of the algorithm. At the same time, the number of iterations is used to control the step size adaptively to ensure that the MRSA has a large τ value at the initial stage of iteration, so it has strong exploration ability. As the number of iterations increases, the τ value decreases gradually, which improves the exploitation ability of the MRSA.

The basic steps of MRSA are as follows:

Step 1: Initialize parameters of MRSA, including the number of candidate solutions (*N*), the dimension size of the search space (*D*), the upper bound (*ub*) and lower bound (*lb*) of the solution, the maximum number of iterations (*T*), and two sensitive parameters (*α* and *β*).

Step 2: Apply Tent mapping to initialize the solutions’ positions. Generate *N* D-dimensional chaotic sequences and carry their components to the value range of spatial variables of the original problem through Equation (10).

Step 3: Calculate the fitness function for the candidate solutions and find the best solution so far. Then, update the *R*, *ES*, *η* and *P* parameters using Equations (4)–(7).

Step 4: According to the relationship between the current number of iterations (*t*) and the maximum number of iterations, two main methods (exploration and exploitation) with four strategies are used to update the position. Candidate solutions use Equation (3) to attempt to expand the search area when t≤T/2 and use Equation (8) to attempt to converge toward the near-optimal solution when t>T/2.

Step 5: After one iteration, calculate the fitness value of each crocodile and find the current optimal individual.

Step 6: Apply the Gaussian random walk strategy to disturb the position of crocodiles. The fitness function value after disturbance is calculated and compared with that before disturbance. If the individual after the random walk becomes better, the candidate solution is changed to the position after the random walk; otherwise, keep the original individual unchanged.

Step 7: Determine whether the MRSA algorithm runs to the maximum number of iterations. If so, jump out of the loop and output the optimization results; otherwise, return to step 3.

#### 2.4.4. MRSA-SVM

**Figure 2:**
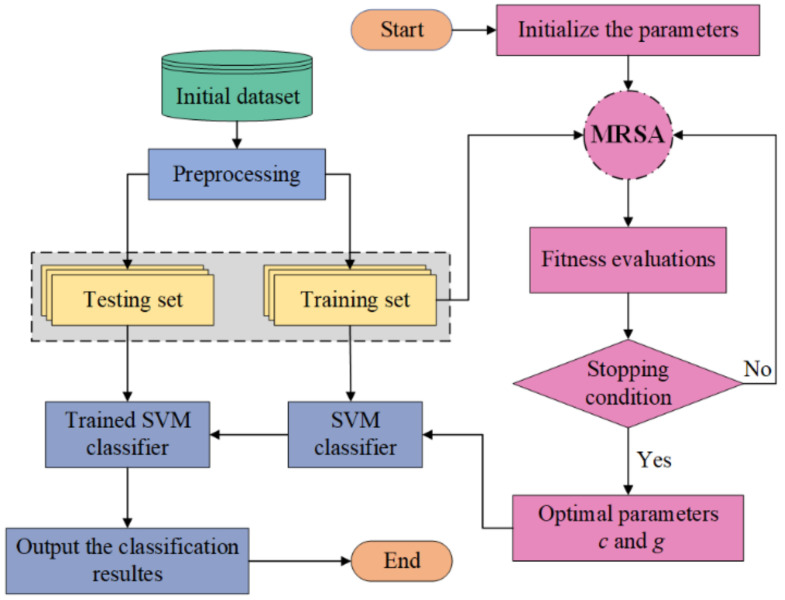
MRSA-SVM flowchart.

Apply the proposed MRSA to optimize the penalty parameter *c* and the kernel parameter *g*. The basic idea of the MRSA-SVM model is that parameters *c* and *g* are regarded as individual position variables of the crocodile population. In the two-dimensional space composed of *c* and *g*, the SVM classification accuracy for five-fold cross-validation of the training set is used as the fitness function to evaluate the individual position. On this basis, we update the location information of the crocodile population by two main methods (exploration and exploitation) with four strategies: high walking, belly walking, hunting coordination and hunting cooperation. MRSA outputs the optimum parameters *c* and *g*, and it transfers them to establish an SVM classification model. The trained SVM model is applied to detect the test set. The MRSA-SVM flowchart is shown in Figure 2.

### 2.5. Evaluation of Model Performance

In addition to the commonly used accuracy as the evaluation index, other evaluation indexes were used for the comprehensive evaluation of model performance, such as recall, precision, F_1_, and kappa coefficient. For all these indexes, values closer to 1 imply a better classification performance of the model. The calculated equations are as follows:

(13) $$\left\{\begin{array}{l} \mathrm{Accuracy}=\frac{\left(TP+TN\right)}{\left(TP+FP+TN+FN\right)}\\ \mathrm{Recall}=\frac{TP}{TP+FN}\\ \mathrm{Precision}=\frac{TP}{TP+FP}\\ F_{1}=\frac{2\times \mathrm{Precision}\times \mathrm{Recall}}{\mathrm{Precision}+\mathrm{Recall}}\\ \mathrm{Kappa}=\frac{p_{0}-p_{e}}{1-p_{e}} \end{array}\right.$$

in which

(14) $$\left\{\begin{array}{l} p_{0}=\frac{TP+TN}{TP+FP+TN+FN}\\ p_{e}=\frac{\left(TP+FP\right)\times \left(TP+FN\right)+\left(TN+FN\right)\times \left(TN+FP\right)}{{\left(TP+FP+TN+FN\right)}^{2}} \end{array}\right.$$

In these equations, *TP* is the true positive (positive jujube samples are detected as positive samples); *FP* is the false positive (negative jujube samples are detected as positive samples); *TN* is the true negative (negative jujube samples are detected as negative samples); and *FN* is the false are detected as negative samples). F_1_ is the weighted average of recall and precision. The kappa coefficient is an indicator of a consistency test that can penalize the preference of the model in the case of an unbalanced sample number. In this study, all of the processes described for the development, optimization, and evaluation of models were implemented in Matlab R2019b (MathWorks, Natick, MA, USA).

## 3. Results

### 3.1. Spectral Characteristics

**Figure 3:**
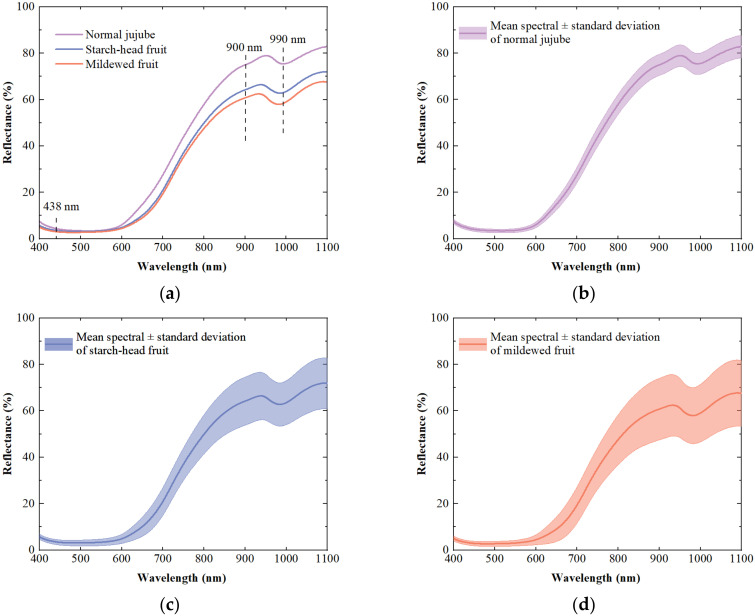
Spectral reflectance curves of dried Hami jujube in different states. (**a**) Mean reflectance spectral curves, and spectral curves with standard deviation for (**b**) normal jujube, (**c**) starch-head fruit, and (**d**) mildewed fruit.

The signal-to-noise ratio at both ends of the spectral data was low, which could not provide effective spectral information. Therefore, spectral bands in the range of 400–1110 nm (934 bands in total) were selected for modeling and analysis. Figure 3 showed the mean spectral reflectance curves and their standard deviations of dried Hami jujube in different states. The similar trends and peak positions come from the common compounds and structures, while the differences should be caused by the changes in chemical components such as moisture and protein during the formation of starch-head and mildewed fruit. In detail, the spectral reflectance in the visible light region of 400–600 nm is relatively low, which may be caused by the strong absorption of light by the pigments in the dried Hami jujube. Approximately in the 600–750 nm spectral range, the reflectance rises sharply, which could be ascribed to the ‘red edge’ of individual organisms [33]. The weak valley near 890 nm corresponded to the third overtone of C-H [34]. Another valley around 990 nm was related to the moisture content of dried Hami jujube and might be associated with the second overtone of O-H [35]. Compared with normal jujube and starch-head fruit, the spectral curves of mildewed fruit had a large deviation distribution, which would be caused by the influence of different mildew degrees. Mold erosion made the mildewed fruit dull in color, attached to mycelium, and deteriorated in pulp. These changes altered the transmission path of light and improved the absorption ability of mildewed fruit to light, resulting in a significant decrease in spectral reflectance. This is in agreement with the findings of other studies [36,37]. The spectral curve of starch-head fruit was between the mildewed fruit and the normal jujube, and it is closer to the mildewed fruit, which further proved that the continuous development of starch-head fruit would become mildewed fruit. The difference of spectra provided a detection basis for the detection of starch-head and mildewed fruit. Unfortunately, the spectral curves of different groups overlapped, and we cannot classify the jujube’s state by spectral information directly. Therefore, spectral data processing and model building were necessary to make an accurate classification.

### 3.2. Comparison of Imbalanced Class Handling Methods

The oversampling methods were applied to solve the data imbalance problem: that is, more starch-head fruit than normal jujubes and mildewed fruit. Table 1 showed the classification accuracy of the models built on datasets generated by different oversampling methods. To better discriminate the effectiveness of oversampling methods, PLS-DA linear models were also established, and the latent variables (LVs) were determined by five-fold cross-validation. It was apparent from this table that the classification effects of SVM models were generally better than those of PLS-DA models. This indicated that the nonlinear relationship between spectral data and classification labels was stronger, and SVM models can handle this relationship better.

The discrimination accuracy of models based on null oversampling training data was unsatisfactory. It was due to the high similarity of spectral data between mildewed fruit and starch-head fruit. In addition, another more important reason was that the data of mildewed fruit was relatively few, which caused the modeling effect to be biased. The accuracy of the models based on the balanced data processed by oversampling methods had been greatly improved, especially for mildewed fruit. The validity of the new synthetic data and its positive effects on modeling were demonstrated. In terms of imbalanced data handling, BL-SMOTE was more efficient than other methods. The overall accuracy of the SVM model increased to 93.33%, and that of the PLS-DA model increased to 88.33%. In particular, the classification accuracy of mildewed fruit increased by 17.24%.

**Figure 4:**
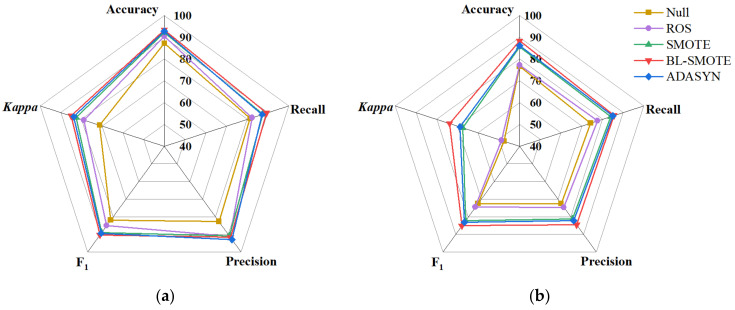
Comprehensive evaluation indexes (%) of test set: (**a**) SVM model; (**b**) PLS-DA model.

In order to comprehensively evaluate the impact of oversampling methods on the model performance, Figure 4 presented the radar charts based on the accuracy, recall, precision, F_1_, and kappa coefficient of the test set. The overall value located in the outermost circle of the radar chart indicated that the comprehensive performance of the model was excellent. The overall distribution of the SVM model was more biased toward the exterior than that of the PLS-DA model, thus indicating that the SVM model was more suitable for the detection of dried Hami jujube. What stood out in the Figure 4 was that the red lines that represented the BL-SMOTE method were at the outermost circle in each dimension, again proving that BL-SMOT outperformed the other methods. The accuracy, recall, precision, F_1_, and kappa coefficient of the test set based on the SVM model established by BL-SMOTE data were 93.33%, 89.34%, 91.64%, 90.34% and 85.00%, respectively. Thus, the SVM model combined with the BL-SMOTE balanced training set was used for subsequent analysis.

### 3.3. Analysis of Variable Selection Strategies

**Figure 5:**
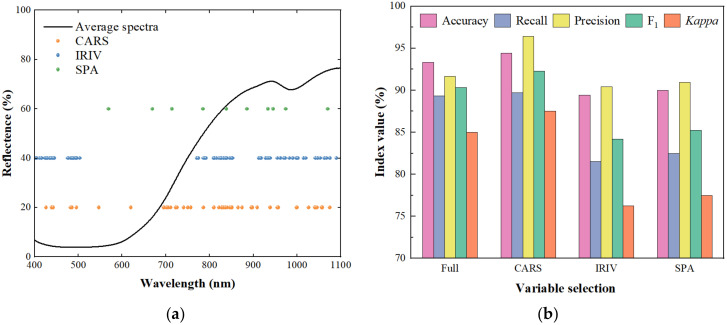
(**a**) Distribution map of feature variables. (**b**) Test set results of SVM model based on these variables.

Figure 5a showed the distribution of feature variables extracted by different variable selection strategies. The number of variables extracted by the three algorithms was significantly reduced compared to the full spectrum. With the CARS, IRIV and SPA algorithms, the number of spectral variables was reduced from 934 to 52, 95 and 12, accounting for 5.57%, 10.17% and 1.28% of the full wavelength, respectively. The distributions of the variables selected by CARS and IRIV were similar. Overall, the selected variables were mostly distributed above 700 nm, which might be related to the stronger penetration ability of the near-infrared. These spectral data contained more information about organic compounds such as moisture, sugar and protein [38,39], which could better characterize the difference between dried Hami jujubes in different states. The extracted variables were used to construct SVM models, and the results of the test set are shown in Figure 5b. Only the proper variable selection strategy can help obtain a more accurate and robust classification performance. In this study, CARS obtained superior results. IRIV might retain too many variables and cause redundancy, while SPA deleted important variables and caused feature loss. After CARS variable selection, the accuracy, recall, precision, F_1_ and kappa coefficient of the test set were 94.44%, 89.69%, 96.44%, 92.26% and 87.50%, respectively.

### 3.4. Establishment of MRSA-SVM

Although the SVM model obtained based on BL-SMOTE and CARS methods can detect dried Hami jujube, the accuracy still has room for improvement. To further improve the classification performance, MRSA was introduced to optimize the parameters of SVM model, and then, it was compared with RSA, GA and PSO. In all experiments, we set the population size to 20 and the maximum number of iterations to 50. The optimization range of penalty parameter *c* and the kernel parameter *g* was set to $\left[2^{-2},2^{8}\right]$. Table 2 showed the results of parameter optimization and the test set. The optimization algorithms improved the classification performance of the SVM model except for GA. Obviously, MRSA-SVM obtained the best detection results.

**Figure 6:**
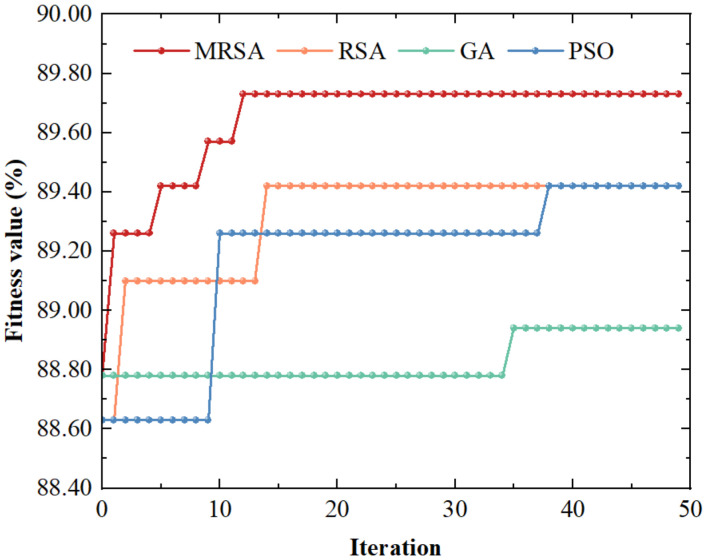
Optimization process of MRSA, RSA, GA and PSO algorithm.

**Figure 7:**
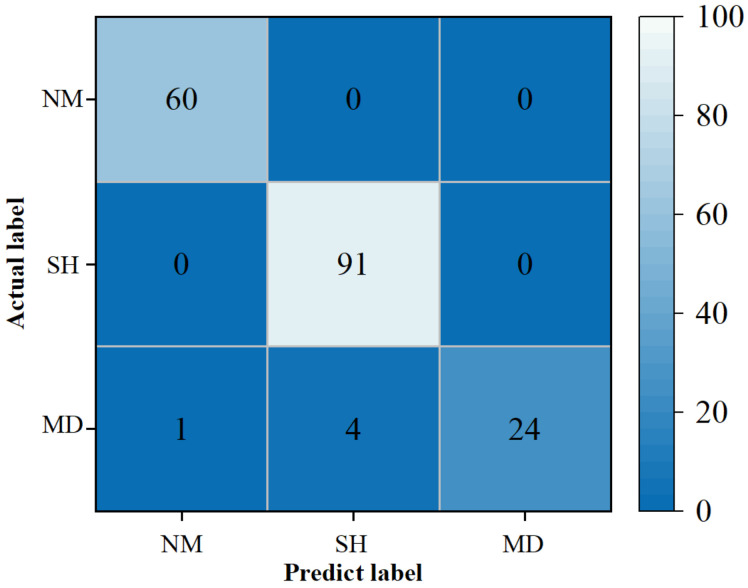
Confusion matrix of test set based on MRSA-SVM. NM, SH and MD represented normal jujube, starch-head fruit and mildewed fruit, respectively.

Figure 6 was the optimization process for the models. In terms of convergence speed, MRSA converged around the 12th generation, which was slightly faster than RSA. Meanwhile, GA and PSO were not converged in the 30th generation. According to the fitness value, MRSA jumped out of the local optimum well and finally stabilized at 89.73%, indicating that MRSA has a stronger search ability. The fitness values of PSO and RSA were stabilized at 89.42%, and there was still room for optimization, namely that they had not jumped out of the local optimum. In addition, MRSA-SVM also performed the best on the test set, which further proved the excellent robustness of the model. Figure 7 was the confusion matrix of the test set based on the MRSA-SVM model. We can see that both normal jujubes and starch-head fruit were accurately discriminated. Among the mildewed fruit samples, one was mistakenly judged as normal jujube, and four were mistakenly judged as starch-head fruit. The overall accuracy of the test set was 97.22%. All results showed that the MRSA-SVM model could be used to detect starch-head and mildewed fruit in dried Hami jujube.

## 4. Discussion

Vis-NIR spectroscopy is an effective method to realize the rapid and non-destructive detection of jujube quality [40,41,42]. Most of these studies have focused on fresh jujubes. Actually, jujubes are mostly marketed in dried form, so it is significant to eliminate defective fruit in dried jujubes. Compared with fresh jujube, the epidermal cuticle of dried jujube was changed, and there were many irregular folds on the surface. These characteristics lead to defects hidden deep that are difficult to identify, which increases the difficulty of non-destructive detection. In addition, there are few reports on the detection of starch-head fruit. The starch-head fruit is between normal jujube and mildewed fruit, and its surface has no obvious defect characteristics, so it is not easy to identify with the naked eye. Starch-head fruit will develop into mildew fruit when it is not removed, causing food safety hazards. In this study, we achieve the rapid detection of starch-head and mildewed fruit in dried Hami jujube to further expand the application range of Vis-NIR spectroscopy. In addition to Vis-NIR spectroscopy, machine vision and hyperspectral imaging are commonly used methods for defective fruit detection [37,43,44]. Machine vision has a high detection rate for defects with obvious changes in external characteristics, but it is not easy to identify defects without obvious characteristics. In this study, the peel of mildewed fruit has plaque, which is quite different from normal jujube and can be identified by the machine vision method. However, for the starch-head fruit, the appearance of the sample is less different from that of the normal jujube, and it is not easy to identify by machine vision. Compared with machine vision, spectral information can reflect the changes in the content and types of compounds in starch-head and mildewed fruit, and further analysis can realize the category discrimination. The hyperspectral imaging integrates machine vision and spectroscopy, which can simultaneously acquire the spatial and spectral characteristics of samples and can detect the internal and external defects of fruits. However, the price of hyperspectral imaging equipment is high, the sampling time is long, and the acquired data have the problem of dimensional disaster. Compared with hyperspectral imaging, Vis-NIR spectroscopy has a fast sampling speed and simple detection method, which is more suitable for enterprise production. The proposed method can provide technical reference for the development of an automatic defect detection device for dried jujubes.

Balanced distribution and representative experimental samples were the keys to construct classification models for dried Hami jujube. Oversampling techniques combined with appropriate machine learning models can solve the class imbalance problem and further improve the classification performance of the model [18]. In this study, BL-SMOTE and ADASYN improved the model significantly, and BL-SMOTE was slightly better. In addition, oversampling methods combined with SVM models outperformed PLS-DA models. ROS randomly replicated the minority class samples, although it can also improve the performance of models, but the effects were not obvious, especially for mildewed jujubes. Both BL-SMOTE and ADASYN were improved algorithms based on SMOTE, which might be the reason why they are better than SMOTE. Although some previous studies [45,46,47] have also adopted oversampling methods, there are few reports on applying multiple oversampling methods to balance spectral data and compare their effects. Our research results can be used as a reference. It is worth mentioning that the optimal oversampling method needs to be determined according to the detection results of the model, combined with specific cases.

A suitable meta-heuristic optimizer can effectively optimize the parameters of the SVM model, thereby improving the classification performance. The previous research results confirmed this conclusion [19,47]. A comparison of the classification results for dried Hami jujube based on different models revealed the superiority of the MRSA-SVM model. Moreover, the fast convergence speed and good fitness value further confirmed the improvement effect of our improvement measures on RSA. Tent chaotic mapping optimized the initial solution [48], while the Gaussian random walk strategy enhanced the exploration ability and improved the local exploitation ability of the algorithm [49]. Other algorithms can also be tried to further optimize the model to obtain better classification results. This study facilitates the non-destructive detection of quality for dried jujubes and other dried fruits, thereby accelerating the development of agro-products.

## 5. Conclusions

In this study, a non-destructive detection method of starch-head and mildewed fruit in dried Hami jujube using visible/near-infrared spectroscopy based on BL-SMOTE and MRSA-SVM was proposed. The BL-SMOTE successfully handled imbalanced spectral data and improve the model performance. Furthermore, the MRSA-SVM using variables extracted by CARS adaptively selected the parameters for defective fruit detection of jujube samples and demonstrates superior classification accuracy compared with other models. High accuracy and evaluation metrics verified the effectiveness of the proposed method. In brief, this method can be used to detect the starch-head and mildewed fruit in dried Hami jujubes, avoiding the hidden dangers of food safety. The established models have the potential for practical application, which is of great significance for dried jujubes’ future quality detection, grading and automatic screening. In addition, these methods could provide technical reference for quality detection of other kinds of dried fruits. In further work, this method will be combined with a microbiological test to species identification and quantitative detection of fungi in mildewed fruit. In addition, other varieties of dried fruits will be added to optimize the robustness and effectiveness of this detection model.

## Figures and Tables

**Table 1:** The discrimination results based on different oversampling methods.

Model	Over-Sampling	Accuracy for Training Set (%)				Accuracy for Test Set (%)			
		NM ^a^	SH ^b^	MD ^c^	Total	NM	SH	MD	Total
SVM	Null	100.00	92.89	82.61	93.57	100.00	89.01	55.17	87.22
	ROS	99.05	95.26	100.00	98.10	96.67	98.90	51.72	90.56
	SMOTE	100.00	95.74	95.74	97.16	98.33	95.60	68.97	92.22
	BL-SMOTE	99.53	94.79	98.10	97.47	100.00	95.60	72.41	93.33
	ADASYN	99.52	94.31	97.60	97.12	98.33	97.80	65.52	92.78
PLS-DA	Null	98.57	72.04	71.01	80.71	98.33	69.23	55.17	76.67
	ROS	97.63	83.41	91.94	91.00	96.67	67.03	68.97	77.22
	SMOTE	96.68	78.20	87.20	87.36	98.33	81.32	72.41	85.56
	BL-SMOTE	98.57	82.00	89.57	90.56	96.67	87.91	72.41	88.33
	ADASYN	98.06	81.52	92.31	90.05	96.67	82.42	75.86	86.11

^a,b,c^ represent normal jujube, starch-head fruit and mildewed fruit in dried Hami jujubes.

**Table 2:** Detection results of test set base on SVM optimized by different algorithms.

Optimizer	*c*	*g*	Accuracy (%)	Recall (%)	Precision (%)	F_1_ (%)	Kappa (%)
MRSA	10.13	5.49	97.22	94.25	98.05	95.86	93.75
RSA	8.34	5.28	96.11	92.74	96.31	94.25	91.25
GA	3.76	12.89	93.33	87.39	95.84	90.32	85.00
PSO	6.48	6.85	95.56	91.40	97.06	93.63	90.00

## Data Availability

The data presented in this study are available on request from the corresponding author.
